# A Sunday’s Dinner

**Published:** 1871-08

**Authors:** 


### A SUNDAY'S DINNER

Is made the most sumptuous meal of the week in a great many households, and the guests retire from the table more like gorged anacondas than intellectual human beings, with the result that during the whole afternoon there is such an amount of mental, physical, and religious sleepiness, if not actual stupidity, that no duties whatever are performed with alacrity, efficiency, and acceptableness. The Sunday dinner made of a cup of hot tea, some bread and butter, with a slice of cold meat, and absolutely nothing else, would be wiser and better for all; it would give the servants more leisure, the appetite would be as completely satisfied half an hour afterwards, while body, brain, and heart would be in a fitting condition to perform the duties of the Sabbath with pleasure to ourselves, with greater efficiency to others, and doubtless with larger acceptance to Him toward whom all our service is due.

